# Mental Health Difficulties in Children who Develop Misophonia: An Examination of ADHD, Depression & Anxiety

**DOI:** 10.1007/s10578-023-01569-y

**Published:** 2023-07-28

**Authors:** Louisa J Rinaldi, Julia Simner

**Affiliations:** School of Psychology, University of Sussex, Brighton, UK

**Keywords:** ALSPAC, DAWBA, mental health, anxiety, depression, ADHD, misophonia

## Abstract

Misophonia is a sound sensitivity disorder characterized by unusually strong aversions to a specific class of sounds (e.g., eating sounds). Here we demonstrate the mental health profile in children who develop misophonia, examining depression, anxiety and ADHD. Our participants were members of the birth cohort ALSPAC (*Avon Longitudinal Study of Parents and Children*). We screened them for misophonia as adults, then analysed their retrospective mental health data from ages 7 to 16 years inclusive, reported from both children and parents. Data from their *Development and Wellbeing Assessments* (7–15 years) and their *Short Mood and Feelings Questionnaires* (9–16 years) show that our misophonia group had a greater likelihood of childhood anxiety disorder and depression in childhood (but not ADHD). Our data provide the first evidence from a large general population sample of the types of mental health co-morbidities found in children who develop misophonia.

Misophonia is a sound sensitivity disorder in which certain classes of sound feel unusually unpleasant [1]. Typical triggers include everyday ambient sounds (e.g., chewing, breathing) which are easily ignored by most other people, but feel highly aversive to people with misophonia (henceforth misophonics). Encountering these sounds causes negative emotions for misophonics, such as anger, disgust, or anxiety, and also physiological reflexes such as increased heart rate, muscle flinch, or feeling of pressure [2]. The strongest triggers of misophonia are human bodily sounds such as eating sounds (e.g., chewing, crunching, slurping), but can also be non-human noises (e.g., clicking, tapping), or even non-auditory repetitive actions such as chair-rocking [3]. Misophonia symptoms have been linked to subtle organisational differences in the brain, including increased functional and structural connectivity in regions related to threat, emotion, and salience [4, 5]. This suggests sounds are more prominent and emotionally salient for people with misophonia, which appears to make them more distressing to hear, and harder to ignore.

In the two decades since misophonia was first named and recognised [6], a small but rapidly-growing body of research has begun to comprehensively analyse this sound sensitivity disorder, identifying common co-morbidities (e.g., obsessive compulsive disorder, autism; [7–9]) and component facets (e.g., emotional symptoms vs. physiological symptoms vs. behavioural symptoms; [10, 11]). However, the vast majority of misophonia studies focus exclusively on adult sufferers. Early exceptions were several case-studies describing children and adolescents with misophonia who were seeking treatment in clinical environments [12–15]. Subsequently, our own study was the first to explore misophonia in randomly-sampled children who had *not* referred for treatment. This provided important information because, unlike children at clinics (who already show sufficient difficulties for their caregivers to seek clinical support), children from randomly-sampled populations demonstrate how misophonia manifests in the wider community. Here we found that average children with misophonia 10–14 years showed significantly poorer wellbeing than their peers, in terms of their life-satisfaction, health-related quality of life, levels of anxiety, obsessive compulsive traits, autism traits, sensory sensitivities, and emotion dysregulation [7, 16]. This work provided the first evidence that misophonia in the wider childhood population brings sufficient difficulties in mental health for it to be an important target for research and clinical support.

Our earlier studies had several important limitations. In seeking a random sample of children with misophonia, we screened 150 children, but this produced a testing group of only 15 target misophonics [16]. Although this was sufficient to detect large effect sizes, a bigger sample would be needed for more fine-grained work. Second, we tested adolescent participants in the later stages of childhood (10–14 years), leaving the period prior to this as yet unexplored (for community misophonics). Third, our data were based on eliciting self-reports from children, which although relatively successful in our previous work, might nonetheless introduce greater noise than when testing adults. Indeed, this drawback is a cornerstone of developmental science, but one we aim to overcome in the current study. Here, we have had access to a unique cohort of adults whose early life has been rigorously and scientifically documented as part of the *Children of the 90s Cohort* of the *Avon Longitudinal Study of Parents and Children* (ALSPAC; [17]. These 30-year-old adults have been screened extensively since before birth (as far back as in utero) on a wide range of topics including, importantly, mental health. In the current study we screened this cohort at the age of 28 years, identifying several hundred participants with misophonia, and several thousand without (to serve as a comparison group). Then, using data originally gathered from these adults when they were still children, we conducted a series of analyses to ask what are the timepoints in development when children in the misophonia group diverged from their peers across a range of mental health variables.

In our study, we examined three mental health conditions in particular: anxiety disorder, depression, and attention deficit hyperactivity disorder (ADHD). Our choice was based on studies of misophonia in adolescents and adults, which show not only that quality-of-life declines with increasing misophonia symptoms [16, 18] but that depressive symptoms increase [19, 20], as do rates of anxiety compared with non-misophonics [8, 16]. Poor mental health also remains a significant predictor of misophonia even after controlling for age and sex (e.g., in anxiety-related panic disorders; [21]). Since these mental health conditions are known to have roots childhood (e.g., three quarters of anxiety disorders originate in childhood; [22]) this leads us to hypothesise that anxiety and depressive traits may already exist in young misophonics. We looked additionally at ADHD tendencies, although our hypothesis here is less clear. One study [18] found that 5% of their 575 adult misophonics had co-morbid ADHD, while a figure reported elsewhere was 12% [23]. However, we have found it hard to evaluate whether these rates differ from the population baseline (of 4.4%; [24]), due either to our uncertainty over their methodology ([23]^1^), or due to their participant-selection criteria, which in one study [18] *a priori* excluded participants with primary ADD/ADHD. This method of participant-selection initially suggests ADHD may be especially high in misophonia, since removing cases of ADHD still left a prevalence similar to the population baseline. Crucially however, their recruitment took place at a misophonia clinic, where the most severe cases are likely to gather. It is therefore unclear whether ADHD would be elevated in the wider general population of people with misophonia, so we address this question here. In the current paper, we tentatively hypothesise that rates of ADHD may be higher in misophonia simply because there is a known link between ADHD and sensory sensitivities [25–28]. This prediction therefore sits alongside our similar hypotheses for elevated depression and anxiety traits.

To give further background to our study, our first measure was the *Development and Wellbeing Assessment* (DAWBA; [29]). It comprises a series of clinical questions to determine the probability of having different mental health conditions at different age-points, including anxiety, depression, and ADHD. We therefore examined data from this measure, completed by parents between 1998 and 2007 (when children were 7–15 years old). By evaluating scores at different ages, we can provide a valuable ‘quasi real-time’ simulation of how and when different mental health conditions emerge for children in our misophonia and comparison groups. In addition, we also compare our two groups on a further measure for depressive traits (the short *Mood and Feelings Questionnaire*, sMFQ; [30]), administered to both children and parents from the age of 9/10 to 16 years. Across all measures, we predict that participants in the misophonia group will show an increased risk of anxiety disorder, depression and possibly ADHD in childhood compared to their peers, and that this is likely to become apparent at least by adolescence. Importantly, our retrospective study cannot identify whether these mental health symptoms are elevated in children *who already had* misophonia at that age, since misophonia tests were not administered to ALSPAC children (and indeed the condition was yet to be recognised for another decade). Instead however, our study can tell us whether mental health symptoms are elevated in children who *go on to report misophonia as adults* - who therefore either manifested it as children, or went on to develop it subsequently.

## Methods

### Participants

Our participants were drawn from the *Avon Longitudinal Study of Parents and Children* (*ALSPAC*) a British longitudinal cohort with a wealth of psychological, genetic, educational and health data spanning three decades [17, 31, 32]. Pregnant women who were resident in Avon, UK with expected dates of delivery from 1st April 1991 to 31st December 1992 were invited to take part in the study. The initial number of pregnancies enrolled was 14,541, with an additional 913 eligible pregnancies added retrospectively in subsequent waves. These 15,454 pregnancies resulted in 15,589 foetuses, of which 14,901 were alive at 1 year of age. In the current study we screened for misophonia almost three decades later in Dec 2020- Feb 2021 as part of the “Life at 28+” wave of data collection, in over 4000 of the active remaining respondents from the index cohort (known as the ‘Children of the 90s Cohort”). Our participants with returned data were 4253 adults aged 28, comprising 1452 males-at-birth (mean age in months 345.81; SD 5.94), 2798 female-at-birth (mean age in months 345.49; SD 6.04) and 3 with no information about sex-at-birth (mean age in months 338.67; SD 4.51). The outcome of our screening for misophonia (see *Materials* for screener) shows that this group contained 333 adults with misophonia (77 male and 256 female) and 3920 without misophonia (1375 male, 2542 female, 3 unknown). We refer to these as misophonics versus non-misophonics respectively (or misophonics vs. the comparison group). Of these individuals, Tables 1 and 2 show how many of these participants also had childhood data for our measures of interest. Table 1 shows the final number of participants in our DAWBA analyses (for ADHD, anxiety, depression), split by timepoints between 7–15 years. Table 2 shows the final participants for our sMFQ analyses for depression, again split by timepoint, but also by whichever person completed the questionnaire (child completed 10–16 years; parent completed 9–16 years).

**Table 1:** Participant numbers for our misophonic and non-misophonic participants, split by age at DAWBA completion. Also shown is a breakdown by sex-at-birth (female, male) with mean age (& standard deviation) in months for each timepoint

Group	Timepoint (approximate age in years)	Total N	N Female	N Male	Mean age in months (SD)
Total	7	3296	2107	1189	91.79 (1.65)
	10	3304	2107	1197	128.54 (1.50)
	13	3215	2039	1176	166.83 (1.91)
	15	2569	1624	944	184.73 (2.88)
Misophonics	7	247	187	60	91.92 (2.01)
	10	238	181	57	128.48 (0.97)
	13	242	182	60	167.05 (2.44)
	15	194	150	44	184.77 (2.75)
Non-misophonics	7	3049	1920	1129	91.78 (1.61)
	10	3066	1926	1140	128.54 (1.53)
	13	2973	1857	1116	166.82 (1.87)
	15	2374	1474	900	184.73 (2.90)

**Table 2:** Participant numbers split by age at sMFQ completion (both child-completed and parent-completed versions). Also shown is a breakdown by sex-at-birth (female, male) with mean age (& standard deviation) in months for each timepoint

Group	Timepoint (approximate age in years)	Total N	N Female	N Male	Mean age in months (SD)
Total Parent-completed	9	3315	2128	1187	116 (1.42)
	11	3169	2034	1134	141 (1.50)
	13	3168	2019	1149	158 (2.00)
	16	2791	1759	1032	202 (4.32)
Misophonics	9	248	186	62	116 (1.62)
	11	241	186	55	141 (1.90)
	13	231	172	59	158 (1.99)
	16	205	158	47	202 (4.24)
Non-misophonics	9	3067	1942	1125	116 (1.40)
	11	2928	1848	1080	141 (1.46)
	13	2937	1847	1090	158 (2.00)
	16	2586	1601	985	202 (4.32)
Total Child-completed	10	3241	2086	1155	127 (2.87)
	12	3154	2026	1128	153 (2.62)
	13	2966	1878	1088	166 (2.35)
	16	2898	1944	954	200 (2.79)
Misophonics	10	254	192	62	128 (2.74)
	12	239	184	55	154 (3.11)
	13	227	174	53	166 (2.69)
	16	224	182	42	200 (2.74)
Non-misophonics	10	2987	1894	1093	127 (2.88)
	12	2915	1842	1073	153 (2.58)
	13	2739	1704	1035	166 (2.32)
	16	2674	1762	912	200 (2.79)

#### Ethical Approval

for the study was obtained from the ALSPAC Ethics and Law Committee and the Local Research Ethics Committees. Informed consent for the use of data collected via questionnaires and clinics was obtained from participants following the recommendations of the ALSPAC Ethics and Law Committee at the time.

### Materials

*The Development and Wellbeing Assessment (DAWBA;* [29]). This parent-completed questionnaire poses a series of clinically-based questions about the child, relating to symptoms indicative of mental health conditions. Parents completed this questionnaire at multiple timepoints, specifically when their child was 7, 10, 13 and 15 years. The DAWBA score indicates the likelihood of being diagnosed with a series of mental health conditions based on the criteria of the *International Classification of Diseases-10* (ICD-10) and the *Diagnostic and Statistical Manual of Mental Disorders fourth edition* (DSM-IV). Our hypotheses led us to consider three mental health conditions, which were available across all four time points: ADHD, depression, and anxiety disorder. For ADHD, there were 22 items, which began with a yes/no question (*Over the last 6 months… do you think your child definitely has some problems with overactivity of poor concentration?*) followed by 21 items on a response scale running from 0 to 2 (e.g., *Have their teachers complained over the last 6 months of problems with poor concentration or being easily distracted? No, or doesn’t apply; A little; A lot*). For depression, there were 17 items, comprising 12 yes/no questions (e.g., *In the last 4 weeks, have there been times when your child has been very sad, miserable, unhappy or tearful?*), as well as two items on a 3-point scale (e.g., *When your child has been miserable, could they be cheered up? Easily; With difficulty/ only briefly; Not at all*) and three items on a 2-point scale (e.g., *Over the last 4 weeks, the period of being really miserable has lasted less than 2 weeks; 2 weeks or more?*). Finally, the DAWBA also indicated the likelihood of clinical diagnosis for anxiety disorder. This comprised a package of interviews identifying one or more anxiety disorder from among generalised anxiety disorder, social phobia, separation anxiety, PTSD, OCD, and specific phobias (e.g., *Over the last 6 months has your child worried excessively on more days than not?*). The full list of items and scoring is described in detail for all conditions at https://www.dawba.info/py/dawbainfo/b4.py (see also[29, 33, 34] ).

The DAWBA is a widely-accepted measure described in over 400 publications, and is well validated in both clinical [35] and epidemiological studies [34]. For example, it shows excellent discrimination between clinic and community samples in rates of diagnosed disorders [29] and substantial agreement between DAWBA and case note diagnoses. It has been translated into at least 20 languages (https://www.dawba.info/py/dawbainfo/b4.py), and has been used by national statistics agencies to survey nationwide child psychiatric morbidities (e.g., the UK National Statistics; [33, 36]). In our own sample we examined test-restest reliability, finding a ‘good’ intraclass correlation coefficient (ICC) of 0.85 for ADHD, a ‘moderate’ ICC for anxiety (0.59) although poorer for depression (0.38; [37]). However, our test-retest period was exceptionally long, spanning almost a decade from 7 to 16 years, whereas the ICC is most often applied to test-retest conducted over substantially shorter intervals [37].

*The Short Mood and Feelings Questionnaire (sMFQ)*. This 13-item measure probes depression symptoms over the two weeks prior to testing (e.g., *In the past two weeks… felt miserable or unhappy?*). Responses are given a 3-point Likert scale from *Not at all* (scored 0), *Sometimes true* (scored 1) or *True* (scored 2), and the test is scored out of 26 with higher scores representing more depressive traits. There were both child- and parent-completed versions of the test. Parents completed this questionnaire when their children were 9,11,13 and 16 years, and the equivalent childhood span for the child/self-completed version was 10, 12, 13 and 16 years. The sMFQ is a validated measure of cognitive and affective depressive symptomology, and can successfully discriminate clinically depressed from general population samples in children 8–16 years [37], and depressed from non-depressed children within the general population (where depression was independently identified with the Diagnostic Interview Schedule depression scale; [38])^2^. We measured test-retest reliability in our sample and found an ICC of 0.66 for child-completed sMFQ, and an ICC of 0.75 for parent-completed sMFQ, being ‘moderate’ and ‘moderate-to-good’ respectively [39].

*Sussex Screener for Misophonia (SSfM)*. We developed and administered this written screener as a measure for adult misophonia. The *SSfM* provided examples of known misophonia triggers (*eating noises*; *throat clearing*; *nasal noises*; etc.) as well as a characterisation of misophonia to which participants agreed or disagreed (i.e., *When sounds (e.g., crunching) consistently cause extreme emotions, like anger, disgust or anxiety*). For those agreeing with misophonia, our screener contained further items probing its severity in disrupting daily life (*not at all*, *very mildly*, *moderately*, *severely*, *very severely*) and additional questions for consideration elsewhere, such as whether the participant had sought clinical support for their misophonia, and when they believe it developed (*early school [up to 11]*; *later school [11 to 18]*, *adulthood [18 + years]*). Misophonics were classified as those agreeing with our characterisation of misophonia, but also rejecting ‘not at all’ for its detriment to daily life (i.e., misophonia must disrupt daily life for participants in our misophonia group, at least to some degree). The remainder (i.e., those without any detrimental misophonia) formed our comparison group.

To have confidence that our screener corrected identifies people with misophonia, we first confirmed it converges with multiple lines of evidence for misophonia. Here, we compared it to evidence gathered from assessments administered to the same cohort, including a clinic audiology visit carried out when the cohort were 11 years old [40]. Although misophonia had not been classified or even named at the time of this earlier clinic, there are clear indicators we can use. For example, adults identified with misophonia using our screener were twice as likely than the comparison group to report a sound sensitivity in the clinic assessment at age 11 years (i.e., “Do you ever experience over-sensitivity or distress to particular sounds?”; 6.5% misophonics vs. 3.5% non-misophonics, *χ*^*2*^ (1) = 4.93, *p* = .026). They were also more than twice as likely to be wearing ear defenders at age 11 to protect themselves from aversive sounds compared to their peers (12.5% vs. 5.05%). Although numbers here were too small to test this statistically (since only 16 misophonics were given this latter question^3^), we can also see evidence of misophonia in other converging traits. Hence, our misophonia group were significantly more likely to dislike eating in the presence of others at age 13 (remembering that the very strongest triggers of misophonia are other people’s eating sounds). Here, 5.10% of misophonics disliked eating with others a little or a lot compared to only 2.80% of the comparison group (Fishers exact *p* = .024). At age 25, misophonics were still significantly less likely to eat with others, and instead spent significantly more time than the comparison group eating alone. For example, 13.82% of misophonics ate alone 5 + times during the last week, compared to only 10.81% of the comparison group. This difference is significant (i.e., using the response-scale running from 0 [i.e., never eating alone] to 4 [i.e., 7 + times eating alone], misophonics had a mean of M = 1.42, SD = 1.24; compared to non-misophonics M = 1.16, SD = 1.16; *t*(254.75) = -3.29, *p* = .002, Cohen’s *d* = 0.23). At the same age, misophonics were also significantly more likely to use sound-distractions at the dinner table (e.g., television playing whilst eating) compared with non-misophonics (i.e., mean days without distraction was 1.00 days per week for misophonics [SD 1.18] vs. 1.18 for non-misophonics, SD = 1.20; *t*(261.97) = 2.13, *p* = .034, Cohen’s *d* = 0.15). Finally, at age 25, people classified by our screener as misophonic were also overwhelmingly more likely to indicate typical inter-personal difficulties expected from misophonia (e.g., “In last 6 months compared to people of the same age, I am easily annoyed by others”); i.e., 71.93% misophonics indicated somewhat or certainly true compared to 49.52% of non-misophonics (with respective means on the full scale scored 0–2 [*not true*, *somewhat true*, *certainly true*] as follows: M = 0.94, SD = 0.71; M = 0.57, SD = 0.63; *t*(174.04) = -6.01, *p* < .001, Cohens *d* = 0.58). Finally, we also selected a feature to examine for *divergent* validity, i.e., a feature expected to show *no* difference for misophonics and non-misophonics. As expected from the literature [16], we found that our misophonia screener was entirely non-predictive of *creative self-concept*, a well-studied self-assessment of one’s own creative ability [41, 42] which correlates with direct measurements of creativity, but is known to show no difference between misophonics and non-misophonics [16]. Hence, using an almost identical question to previous misophonia literature (“How good are you at art?”; rated from *not good at all* to *very good*) we found that the creative self-concept of misophonics (M = 3.67, SD = 1.18) was no different to the comparison group (M = 3.57, SD = 1.08; *t*(289.23) = 1.23, *p* = .220, Cohen’s *d* = 0.09), thereby providing divergent validity for our measure of misophonia.

In summary, our screener has both convergent and divergent validity, since participants identified as having misophonia were significantly more likely to have reported a sound sensitivity at age 11, were significantly more likely to avoid eating near others by parental report at 13, and again by self-report at age 25, and were significantly more likely to use sound-distractions while eating (e.g., tv playing). They were also more likely to have been annoyed by others in the 6 months previously, and they explicitly endorsed a careful description of misophonia at the age of 28, while agreeing that it impacted their lives. Finally, as expected, they were no different to the comparison group in their creative self-concept. This ample convergent and divergent validity for our measure leads us to conclude that our screener successfully detects adults with misophonia.

### Procedure

Our SSfM (misophonia screener) was administered as part of ALSPAC’s “Life at 28+” wave of data collection. Participants completed the screener online in digital form, with a pencil-and-paper version made available where requested. The screener took approximately 5-minutes to complete. These data were collected and managed by the ALSPAC team using Redcap, a secure web-based software platform hosted at the University of Bristol, designed to support data capture for research studies [43]. We subsequently accessed our data, alongside existing data from the DAWBA and the sMFQ, which were shared from ALSPAC’s back-catalogue of data from the 1990-2000 s. ALSPAC’s fully searchable data dictionary and variable search tool can be found online at http://www.bristol.ac.uk/alspac/researchers/our-data.

*Analytic Plan*. We examined longitudinal data from the DAWBA (i.e., probability of meeting the diagnostic criteria for ADHD, anxiety disorder and depression) and the sMFQ (symptoms of depression). The DAWBA has an ordinal scale with unequal distances between scale points (e.g., 1 = < 0.1% probability of diagnosis, 2 = ~ 0.5% probability of diagnosis, 3 = ~ 3% probability of diagnosis etc.). We therefore applied a non-parametric test using the *nparLD* package in R [44] which produces ANOVA-type statistics (and also Wald-type statistics, which held the same interpretation for all our results). We present our ANOVA statistics below, henceforth referred to as non-parametric ANOVA. Our models examine differences between groups (misophonics vs. non-misophonics) across four timepoints (7, 10, 13 and 15 years), for three different diagnoses (anxiety disorder, ADHD, depression). Hence we perform three separate analyses, with the outcome being diagnosis likelihood of anxiety disorder, ADHD, and depression, respectively. While ADD/ADHD symptoms decrease with age and are higher in males [45], both anxiety and depression increase with age around the middle of adolescence, and start to emerge more strongly in females from around age 13 [46, 47]. These periods correspond to the upper tail of our age range, so we therefore additionally added sex-at-birth as a predictor (which was provided in our data as male/female). We remind the reader that effect sizes cannot be directly measured for non-parametric mixed ANOVAs [48]. Although some mental health domains also have sub-conditions (e.g., anxiety disorder includes sub-conditions of social anxiety, separation anxiety, specific phobia etc.) we look at domain-level diagnosis. For example, we examine the likelihood of developing anxiety disorder, whether that be social anxiety, separation anxiety, or specific phobias etc. We took this approach following earlier literature e.g., [49] and because fine-grained analyses would be under-powered due to the relative rarity of specific sub-conditions during the period we examine (7–16 years). Finally, we use Mann Whitney Wilcoxons to explore pairwise post-hoc comparisons on ordinal data, correcting for multiple comparisons.

Scores from the sMFQ (depression traits) are continuous but due to non-normality we also ran non-parametric ANOVA using the same framework as before. We analysed child-completed and parent-completed sMFQ scores in separate analyses since agreement between child and parent reports can sometimes be rather low [50, 51]. For the child-completed model, we examined differences between groups (misophonics vs. non-misophonics) across four timepoints (10, 12, 13 and 16 years) and there were also four timepoints for our parent-competed model (9, 11, 13 and 16 years) 4. All analyses were performed in R 3.6.3 using R Studio, with the R packages *tidyverse* for general data wrangling, *ggplot2* for figures, *nparLD* for non-parametric longitudinal analyses.

## Results

### ADHD Diagnosis likelihood - DAWBA

To remind the reader, we ran a non-parametric ANOVA crossing group (misophonic vs. non-misophonic), sex-at-birth (male vs. female), and timepoint (7, 10, 13, 15). Our statistical results are displayed in Table 3 and they show no significant main effect of group (Misophonics M = 0.40, SD = 0.74; Non-misophonics M = 0.35, SD = 0.73), and no significant main effect of time. The effect of sex-at-birth strongly trended (*p* = .052) with females (M = 0.29, SD = 0.64) scoring lower than males (M = 0.47, SD = 0.84) – as expected from the literature [52]. There were no significant interactions between any of our variables. This suggests our misophonia group were no different than the comparison group in their likelihood of an ADHD diagnosis, and had the same trajectory.

**Table 3:** Non-parametric ANOVA showing main effects and interactions between group (misophonic vs. non-misophonic), sex-at-birth (male vs. female) and timepoint (7, 10, 13, 15 years) in predicting diagnosis likelihood of ADHD, using the DAWBA.

	Statistic	Df	p-value
Group	0.56	1	0.453
Sex-at-birth	3.75	1	0.052†*
Time	2.19	2.94	0.088
Group: Sex-at-birth	0.92	1	0.338
Group: Time	1.45	2.94	0.226
Sex-at-birth: Time	0.55	2.94	0.645
Group: Sex-at-birth :Time	1.96	2.94	0.118

Significance level: *** 0.001, ** 0.01, * 0.05, †* strongly trended, missed conventional alpha at third decimal place.

### Anxiety Diagnosis likelihood - DAWBA

**Fig. 1:**
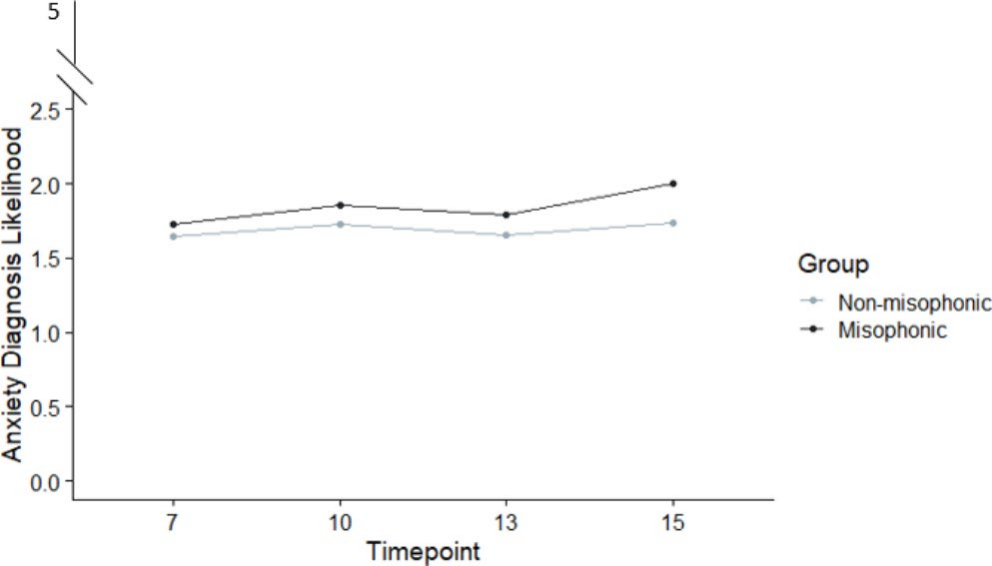
DAWBA-Anxiety scores for misophonics and non-misophonics across four timepoint (7, 10, 13, 15 years). Group and time were both significant main effects, with an interaction at the conventional threshold (see Table 4). Sex-at-birth was also significant l but there was no interaction with group, so our figure reliably shows the pattern of results for misophonia over time

We repeated our analysis (non-parametric ANOVA crossing group, sex-at-birth, and timepoint), but this time looked at the likelihood of a diagnosis of anxiety disorder. The pattern of results was rather different (see Table 4). As expected [46], there was a significant main effect of timepoint, suggesting children became more anxious over time regardless of group status, and there was also a significant main effect of sex-at-birth, since females (M = 1.73, SD = 0.66) were more anxious than males (M = 1.62, SD = 0.61). Our data also showed a significant interaction of sex-at-birth and time, since – again as expected -- males and females show different anxiety trajectories as they age (females typically diverge from boys age 13 years; [46]). Importantly, we also found a significant main effect of group, with misophonics (M = 1.82, SD = 0.66) more anxious than non-misophonics (M = 1.68, SD = 0.64; See Fig. 1).

There was no three-way interaction (group, timepoint, sex-at-birth), and the two-way interaction between group and time approached significance at *p* = .052. Given the interest in age of onset, the two earliest timepoints at age 7 and 10 were explored with non-parametric Mann Whitney Wilcoxon tests. These showed no significant difference between misophonics and non-misophonics at age 7 (*W* = 357,228, *p* = .128, *r* = .02), but a significant difference by the age of 10 years (*W* = 327,241, *p* =. 001, *r* = .05) which survives at the corrected alpha (*α* < 0.025) for multiple comparison. Thus we find evidence that misophonics differ at least from the age of 10.

**Table 4:** Non-parametric ANOVA showing main effects and interactions between group (misophonic vs. non-misophonic), sex-at-birth (male vs. female) and timepoint (7, 10, 13, 15 years) in predicting diagnosis likelihood of an anxiety disorder, using the DAWBA.

	Statistic	Df	p-value
Group	14.20	1	< 0.001***
Sex-at-birth	9.05	1	0.002**
Time	4.19	2.76	0.007**
Group: Sex-at-birth	0.00	1	0.993
Group: Time	2.65	2.76	0.052†*
Sex-at-birth: Time	2.89	2.76	0.038*
Group: Sex-at-birth :Time	0.68	2.76	0.552

Significance level: *** 0.001, ** 0.01, * 0.05, †* strongly trended, missed conventional alpha at third decimal place.

### Depression Diagnosis likelihood - DAWBA

**Fig. 2:**
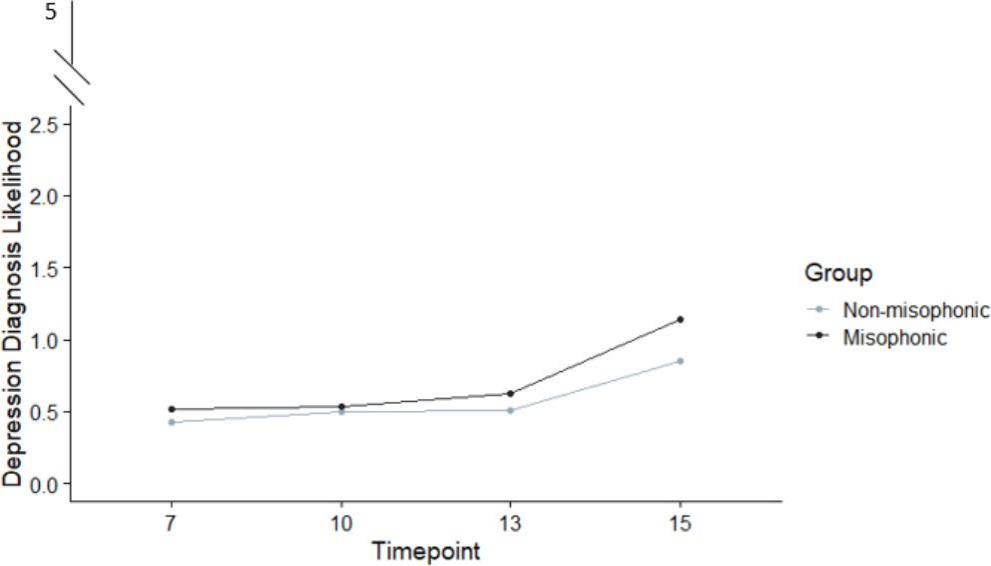
DAWBA-Depression scores for misophonics and non-misophonics across four timepoint (7, 10, 13, 15 years). Group and time did not interact, and were both significant main effects (the former just at threshold; see Table 5). Again, sex-at-birth was significant but there was no interaction with group, so our figure reliably shows the pattern of results for misophonia over time

Our final DAWBA scale was for depression, and again we ran a non-parametric ANOVA crossing group (misophonic vs. non-misophonic), sex-at-birth (male vs. female) and timepoint (7, 10, 13, 15). Our data is represented in Fig. 2, and our final model in Table 5 shows a significant main effect of time and sex-at-birth since mental health symptoms again worsen with age, and are higher for females (M = 0.63, SD = 0.79) than males (M = 0.49, SD = 0.69). There was no significant interaction between time and sex-at-birth, and no three-way interaction with group. In our main comparison of group, misophonics scored higher in their depression-likelihood (M = 0.67, SD = 0.86) than non-misophonics (M 0.57, SD = 0.75) but this just missed significance at a third decimal place (*p* = .054). This raises the question of whether we can replicate this finding at conventional significance in our second depression measure below (i.e., the sMFQ).

**Table 5:** Non-parametric ANOVA showing main effects and interactions between group (misophonic vs. non-misophonic), sex-at-birth (male vs. female) and timepoint (7, 10, 13, 15 years) in predicting diagnosis likelihood of a depression disorder, using the DAWBA.

	Statistic	Df	p-value
Group	3.73	1	0.054†*
Sex-at-birth	11.24	1	0.001***
Time	32.81	2.86	< 0.001***
Group: Sex-at-birth	0.02	1	0.878
Group: Time	0.28	2.86	0.831
Sex-at-birth: Time	1.67	2.86	0.174
Group: Sex-at-birth :Time	1.23	2.86	0.274

Significance level: *** 0.001, ** 0.01, * 0.05, †* strongly trended, missed conventional alpha at third decimal place.

### Depression Symptoms - Parent Completed sMFQ

To remind the reader, we ran a non-parametric ANOVA looking at group (misophonic vs. non-misophonic), timepoint (9, 11, 13, 16 years), and sex-at-birth (male vs. female). Our final model is presented in Table 6 and shows largely the same pattern of results as we saw in our previous depression measure (see DAWBA above).

**Fig. 3:**
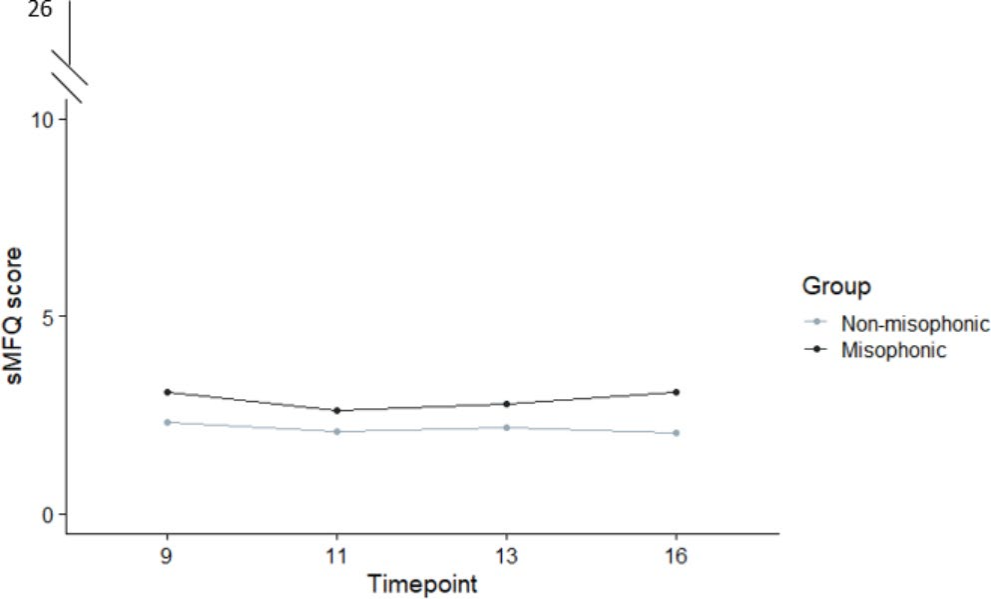
Parent-completed sMFQ-Depression scores for misophonics and non-misophonics across four timepoint (9, 11, 13, 16 years)

As before, there was a significant main effect of time, suggesting that participants’ depressive symptoms worsened with age regardless of group-status. And again as before, we found the expected main effect of sex-at-birth (female M = 2.37, SD = 3.27; male M = 1.96, SD = 2.85) but additionally found a significant interaction between sex-at-birth and time, indicating a sharper trajectory for females. Importantly, we again found a main effect of misophonia, and this was highly significant, showing that misophonics (M = 2.88, SD = 3.67) had significantly more depressive symptoms than non-misophonics (M = 2.17, SD = 3.08; See Fig. 3). There were no two-way or three-way interactions involving group, suggesting that children in the misophonia group were already more depressed than their peers at the earliest age (i.e., by age 9) and were equally affected regardless of their sex-at-birth.

**Table 6:** Multilevel growth model showing main effects of group (misophonic vs. non-misophonic, time (9, 11, 13, and 16 years), and sex-at-birth (male vs. female) as well as the interaction between group and time predicting depressive symptoms via the parent-completed sMFQ (Short Mood and Feelings Questionnaire)

Fixed Effects	Statistic	Df	*p-value*
Group	6.79	1.00	0.009**
Sex-at-birth	7.90	1.00	0.004**
Time	7.47	2.73	< 0.001***
Group: Sex-at-birth	0.24	1.00	0.625
Group: Time	0.21	2.73	0.875
Sex-at-birth: Time	4.04	2.73	0.009*
Group: Sex-at-birth :Time	0.16	2.73	0.909

Levels of significance: *** 0.001, ** 0.01, * 0.05

### Depression Symptoms - Child Completed sMFQ

**Fig. 4:**
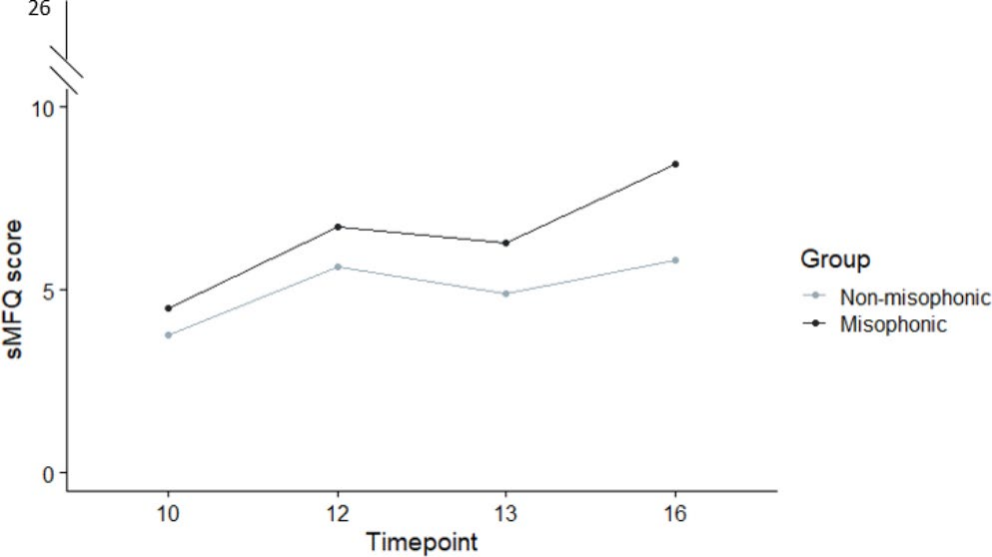
Child-completed sMFQ-Depression scores for misophonics and non-misophonics across four timepoint (10, 12, 13, 16 years)

We repeated our analysis on the child-generated data, which this time took its timepoints at age 10, 12, 13, and 16 years. Our model presented in Table 7 shows an identical pattern of results as we found in parents; i.e., a significant main effect of time and sex-at-birth (females M = 5.53; SD = 4.76; males M = 4.37, SD = 3.71), with a significant interaction between the two. Importantly, we again found a main effect of group, with children in our misophonic group showing greater depressive traits (M = 6.43, SD = 5.11) than our non-misophonic group (M = 5.01, SD = 4.38; see Fig. 4).

**Table 7:** Multilevel growth model showing main effects of group (misophonic vs. non-misophonic, time (10, 12, 13, 16 years), and sex-at-birth (male vs. female) as well as the interaction between group and time predicting depressive symptoms via the child-completed Short Mood and Feelings Questionnaire

Fixed Effects	Statistic	Df	p-value
Group	12.62	1.00	< 0.001***
Sex-at-birth	32.71	1.00	< 0.001***
Time	49.23	2.61	< 0.001***
Group: Sex-at-birth	2.90	1.00	0.089
Group: Time	0.77	2.61	0.492
Sex-at-birth: Time	18.69	2.61	< 0.001***
Group: Sex-at-birth :Time	0.45	2.61	0.693

Levels of significance: *** 0.001, ** 0.01, * 0.05

## Discussion

Our study examined a longitudinal sample from Southern England (ALSPAC; [17]) to test whether adults with the sound sensitivity of misophonia experience mental health difficulties as children. We administered a screener to our sample at the age of 28 years to identify those with misophonia, and then we examined their catalogue of back-data from when they were children. We looked specifically at three mental health conditions (ADHD, anxiety, depression) elicited from two types of measure – the DAWBA for all three conditions, and the sMFQ for depression only. The former was completed by parents when their children were 7, 10, 13, and 15 years. The latter was completed by parents when their children were 9, 11, 13, and 16 years, and was completed by children themselves at ages 10, 12, 13, and 16. Although there was no evidence that children in the misophonia group were unduly impacted by ADHD, there was a clear picture of mental health difficulties for both depression and anxiety. For depression, our data suggest that children in the misophonia group had significantly higher depression symptoms in the sMFQ, and we also tested their likelihood of a depression diagnosis using the DAWBA. The latter showed a near-significant trend (*p* = .054) while the former comfortably passed the conventional alpha threshold, for both adult-report and child-report, providing important replications from both parent and child perspectives. Group status did not interact with other variables suggesting girls and boys with misophonia were equally affected and, crucially, that differences were apparent even at the very youngest age points -- which was ages 9 and 10 respectively for our parent- and child-completed sMFQ (and age 7 for our trending main effect in the DAWBA).

We also found further evidence of mental health difficulties, this time in anxiety: children in the misophonia group scored significantly higher than their peers in their likelihood of an anxiety diagnosis using the DAWBA. In our data, we were indifferent to the nature of the anxiety disorder, meaning that children in the misophonia group scored higher in their likelihood of *any* anxiety condition whether that be social anxiety, generalised anxiety disorder, and so on (with girls and boys in our misophonia group equally affected). Our data indicate differences emerging at a young age - either at age 7 or at age 10, with uncertainly arising since our interaction with time was detected at *p* = .052. A statistically conservative interpretation (rejecting the interaction, leaving only the main effect) is that misophonics diverge at age 7. But a *scientifically* conservative interpretation (accepting the interaction and exploring with post-hoc tests) suggests a difference from age 10. Future studies might therefore further explore the time-course in the development of anxiety, or indeed use additional measures to detect anxiety in children with misophonia at yet younger ages.

In addition to showing mental health developments in misophonia, we also provided convergent and divergent evidence for our screener[40]. Adults identified with misophonia at 28 years had exactly the profile we might expect, including the interpersonal difficulties associated with misophonia, significant early sound sensitivities, and repeated evidence of avoiding communal eating in childhood and adulthood, or taking steps to mask the noise (remembering that the very strongest triggers of misophonia are other people’s eating sounds; [3]). Finally, as expected, they were no different in our divergent measure (of creative self-concept; [16]). This ample convergent and divergent validity leads us to conclude that our screener successfully detected adults with misophonia.

Our data provide a crucial missing link in the misophonia literature, which had pointed strongly to the possibility of specific mental health difficulties in childhood prior to adolescence, but thus far had no direct evidence aside from clinical case studies. From the adult literature we knew that misophonia is associated with elevated rates of both anxiety and depression [8, 16, 19]. This hinted strongly at childhood difficulties since three quarters of adult anxiety disorders, for example, originate in childhood (e.g., [22]). We point out that misophonics also have traits *co-morbid* with anxiety, such as greater rates of autism [53] and also poorer well-being in adolescence [16]. All these facts suggested the possibility of specific mental health difficulties as children, which our data now support.

Our findings are important because anxiety disorder, for example, is among the most problematic mental health disorders in children [54], and early intervention is known to significantly improve life-outcomes (e.g., [55]. Our data suggest that recognising misophonia in children should therefore routinely be interpreted as indicating risk factors for anxiety and depression. Hence where children show aversions to sound (or indeed any collateral traits of misophonia shown here, such as avoiding eating with others or showing a consistent desire to introduce background noise), then this may signal an opportunity to conduct risk assessments for anxiety and depression (e.g., is the child missing school? disengaged socially?). Using misophonia as an indirect pointer to anxiety and depression might also be particularly important because children are less able to verbalise their poor mental health compared to adults [22]. In summary, we propose that recognising misophonia can be extrapolated to recognising the possibility of its co-morbidities in anxiety and depression, and being mindful of the negative consequences these can engender.

In presenting our findings, it is important that we recognise not only our papers’ strengths, but also its limitations. One strength of our paper was that we assessed the ALSPAC cohort for misophonia in 2021, when they were already adults. Doing this allowed us to identify those with and without misophonia in a relatively robust way because we can be more confident in the answers of adults than we might otherwise be had we tested for misophonia at age 7. However, one limitation from this is that we cannot be sure whether those same individuals already had misophonia at the age of 7, when we began our investigation of mental health. It is theoretically possible that this might explain why one effect was non-significant, i.e., parental report of ADHD. However, we suggest that this absence more likely reflects our far weaker a priori hypotheses for ADHD (see *Introduction*). Nonetheless, we present our study in the full understanding that misophonia may not yet have emerged in all the children who went on to present with misophonia as adults. If so, our significant findings will have emerged for one of three reasons. One interpretation is that our significance simply survived the ‘noisy’ environment of a group where some children may not yet have developed the misophonia that would affect them in later life. But a second interpretation is that the mental health difficulties we found are associated with the *disposition* for misophonia, whether or not that misophonia has already emerged. A third interpretation is that misophonia emerges as a *consequence* of these mental health differences, and not as their cause. We are unable to answer this question with the data at hand, and suggest that such data would require large-scale screening of a very large sample of children with a validated diagnostic in order to detect and identify those with misophonia, to follow them in real-time. A power analysis based on our anxiety findings suggests this would require n = 1034 children at 80% power, assuming misophonia is found in at least 10% of cases [16]. Since we have done similar large-scale screening with other sensory differences (e.g., [56]) we do not under-estimate the challenges this would pose, and therefore recognise both the limitations and strengths of our own data.

A further limitation of our study is that our data suggest elevated symptoms of poor mental health but do not represent diagnoses. For example, our DAWBA presents the *likelihood* of a mental health diagnosis from parental report, rather than an actual diagnosis [29]. Similarly, our analysis of the sMFQ shows elevated *traits* rather than clinical diagnostic differences. Hence our results do not indicate whether children in the misophonia group (who certainly had poorer mental health according to parents) did in fact reach the threshold for clinical anxiety or depression at any point in their childhood. Instead, our data simply tell us that their tendency in this direction was elevated. Nonetheless, it is possible to apply caseness thresholds from the literature for our measures, and there are certainly indicators towards a meaningful clinical difference in our misophonia group. In depression, for example, a threshold of 11 in the sMFQ can be applied in line with previous research [57, 58]. When classifying individuals about this threshold (separately for males and females), we found significantly more females passing the threshold in our misophonia group (25.18%; ns = 35 vs. 139) compared to our non-misophonia group (16.95%; n = 274 vs. 1457, 𝜒^2^ = 3.91, *p = .*048), although a smaller and non-significant difference for males (misophonia group 10.42%, n = 5 vs. 48; vs. non-misophonia group 7.03%; n = 68 vs. 967; 𝜒^2^ = 0.67, *p* = .42). As such, levels of depression experienced by children in our misophonia group were not only higher, but more likely to exceed diagnostic thresholds - at least for females. Our results should therefore not be considered insignificant, even if we could not reliably determine clinical diagnoses. Overall, however, the limitations discussed here mean our findings should be taken as preliminary data pointing to the types of mental health difficulties in children who go on to develop misophonia.

## Summary

I﻿n summary, our data show differences in the mental health of children who grow up to manifest misophonia by adulthood. As children, they showed significantly higher scores - firstly in our measures of depression, with differences emerging certainly as young as 9 years (parent-completed sMFQ) and potentially as young as 7 (at a near-significant level using the DAWBA). And in anxiety, children in the misophonia group diverged from their peers certainly as young as 10 (if we accept our threshold interaction) but otherwise from as young as 7 years. Our results bolster what is currently a relatively modest amount of science concerning childhood misophonia, and highlight a need for further research. We also suggest that any future research might be accompanied by actions to widen the public’s understanding of misophonia. To promote wider understanding in our own way, we have created an online information hub (www.misophonia-hub.org) as a one-stop resource containing advice and support for parents, children, educators, researchers and clinicians. In summary, our study shows that misophonia can be identified in adults, and then used to retrospectively inspect the lives of those same individuals when they were children. Doing so revealed that children who later develop misophonia already showed important difficulties in their childhood mental health, with stronger traits in depression and anxiety compared to peers.

## Data Availability

The data in this article is from The University of Bristols’ birth cohort Avon Longitudinal Study of Parents and Children. Requests for access to this dataset should be directed to them directly, please see http://www.bristol.ac.uk/alspac/researchers/.

## Abbreviations

ALSPAC: Avon Longitudinal Study of Parents and Children
DAWBA: Development and Wellbeing Assessment
SSfM: Sussex Screener for Misophonia
sMFQ: Short Mood and Feelings Questionnaire

## Footnotes

1 The prevalence of ADD/ADHD cited in this study is 12%, though we are unclear whether it applies to (i) 12% of their entire sample of 301 misophonics (ii) 12% of the 150 misophonics who reported a co-morbidity, or indeed (iii) ADHD in both misophonics *and* their wider family, or just misophonics alone. Their report reads as follows: “We asked [our 301] participants if they had been diagnosed with other conditions… We also inquired about the presence of neurological, psychological, or psychiatric conditions in their family members. A total of 151 (50%) participants reported no other conditions or diagnoses. From the list of conditions, the most commonly reported items were tinnitus (12%), PTSD (12%), and ADD/ADHD (12%)” (p. 18). Hence, although this earlier study plays an important role in raising the question of ADD/ADHD in misophonia, we hope to contribute further data in the current study.

2 Child-completed sMFQ continued into adulthood though our interest is childhood development, so our age ranges were 9, 11, 13 and 16 years (parent-completed) and 10, 12, 13 and 16 years (child-completed). For evidence on depression and anxiety in adult misophonia we point the reader to the extensive literature (for review see [16]).

3 This n is small because this question appeared in a pipeline within conditional logic, where it was not asked unless earlier questions had been asked and answered in a certain way (i.e., children were asked questions about whether they were over-sensitive to sounds, and only those who replied yes were asked further about ear defenders). However, we include this descriptive statistic for transparency so that every measure we inspected is reported here, even where small numbers meant we did not and could not achieve significance.

4 Data such as ours are superficially amenable to growth model analyses, but our data fail to meet one important assumption of the growth curve model, which is that all individuals follow the same growth pattern [59]. We are likely to violate this assumption since the misophonia we detect in adults will have emerged at varying points throughout their different childhoods. This makes it less likely that all individuals will follow the same growth, and when this assumption is violated, fitting the growth curve model results in less power [59]. We therefore follow Liu and colleagues in applying an ANOVA as most appropriate to our data.
